# Regular use of a hand cream can attenuate skin dryness and roughness caused by frequent hand washing

**DOI:** 10.1186/1471-5945-6-1

**Published:** 2006-02-13

**Authors:** Günter Kampf, Joachim Ennen

**Affiliations:** 1 Bode Chemie GmbH & Co., Scientific Affairs, Melanchthonstr. 27, 22525 Hamburg, Germany; 2 Institut für Hygiene und Umweltmedizin, Ernst-Moritz-Arndt Universität Greifswald, Walther-Rathenau-Str. 49a, 17489 Greifswald, Germany; 3 Beiersdorf AG, Research & Development, Test Centre, Unnastr. 48, 20245 Hamburg, Germany

## Abstract

**Background:**

Aim of the study was to determine the effect of the regular use of a hand cream after washing hands on skin hydration and skin roughness.

**Methods:**

Twenty-five subjects washed hands and forearms with a neutral soap four times per day, for 2 minutes each time, for a total of two weeks. One part of them used a hand cream after each hand wash, the others did not (cross over design after a wash out period of two weeks). Skin roughness and skin hydration were determined on the forearms on days 2, 7, 9 and 14. For skin roughness, twelve silicon imprint per subject and time point were taken from the stratum corneum and assessed with a 3D skin analyzer for depth of the skin relief. For skin hydration, five measurements per subject and time point were taken with a corneometer.

**Results:**

Washing hands lead to a gradual increase of skin roughness from 100 (baseline) to a maximum of 108.5 after 9 days. Use of a hand cream after each hand wash entailed a decrease of skin roughness which the lowest means after 2 (94.5) and 14 days (94.8). Skin hydration was gradually decreased after washing hands from 79 (baseline) to 65.5 after 14 days. The hand wash, followed by use of a hand cream, still decreased skin hydration after 2 days (76.1). Over the next 12 days, however, skin hydration did not change significantly (75.6 after 14 days).

**Conclusion:**

Repetitive and frequent hand washing increases skin dryness and roughness. Use of a hand cream immediately after each hand wash can confine both skin dryness and skin roughness. Regular use of skin care preparations should therefore help to prevent both dry and rough skin among healthcare workers in clinical practice.

## Background

Irritant contact dermatitis is one of the most common occupational disease among health care workers (HCW) [1-3]. A point prevalence of hand dermatitis of 17% – 30% can be found [4,5] which appears to be much higher compared with the general population with a point prevalence of 5.4% [6]. This difference between different professional groups is supported by a retrospective study of Smit & Coenraads [7], in which an overall incidence of 6.5 cases/1000 person-months in nurses and 1 case/1000 person-months in office employees was estimated. The consequences are serious because many employees lose their job due to this hand dermatitis (occupational skin disease): In a population-based register study of occupational skin diseases in Northern Bavaria, Dickel et al. [8] observed an annual incidence rate of 7.3 cases per 10,000 HCW.

The predominant mechanisms of irritation among HCW are frequent wet work, work with occlusive gloves and contact to aggressive surface disinfectants [8,9]. Even water on its own is an irritant [10]. These risk activities often lead to a subclinically impaired skin barrier, before the first clinical irritations (often in the interdigital spaces) become visible [11,12]. The typical clinical picture is a combination of rough, dry and scaly skin, erythema and a burning sensation [1]. In daily routine, HCW are exposed to both: washing of hands and disinfection with alcohol-based hand rubs. Commonly used alcohol-based hand rubs contain emollients and are usually well tolerated on intact skin [13,14]. If the epidermal barrier becomes disrupted and alcohol causes that burning sensation during use, this is often interpreted by the user as "aggressiveness" of the alcohol-based hand rubs. As a logical consequence, the user reduces the applications with alcohol-based hand rubs and tries to compensate it with increased hand washing procedures. This leads unfortunately rather to an increase of the barrier disruption, which is for a while unnoticed, but will often lead to clinically relevant hand dermatitis. A vicious circle is initiated. That is why the use of skin care preparations is clearly recommended to minimize the occurance of irritant contact dermatitis associated with hand antisepsis or hand washing [15]. But the effect of skin care preparations under such conditions has so far never been studied [16]. That is why we have looked at the effect of frequent, thorough and repetitive hand washing with and without regular use of a cream after each wash on skin roughness and skin hydration as the principal clinical symptoms of irritant contact dermatitis.

## Methods

### Study subjects

25 human study subjects after giving their informed consent were included. They had no apparent skin disease on hands and forarms. Based on a clinical assessement of each volunteers' skin, fourteen subjects were classified to have rather dry skin. The remaining eleven subjects were found to have normal skin.

### Test preparations

For washing hands a standard liquid soap was used containing sodium tallowate, sodium cocoate, sodium palm kernelate, aqua, glycerin, paraffinum liquidum, perfume, octyldodecanol, sodium chloride, prunus dulcis, sodium thiosulfate, tetrasodium etidronate, lanolin alcohol, disteardimonium hectorite, linalool, hexyl cinnamal, alpha-isomethyl ionone, coumarin, benzyl salicylate, limonene, butylphenyl, methylpropional, hydroxylisohexyl 3-cyclohexene carboxaldehyde, and CI 77891. Baktolan cream was used in one part of the subjects for treatment of hands and forearms after each hand wash. It contains aqua, mineral oil, petrolatum, paraffinum liquidum, ozokerite, glycerin, glyceryl oleate, lanolin alcohol, microcrytalline wax, montan wax, BHT, ascorbyl palmitate, glyceryl stearate, citric acid, propylene glycol, magensium sulfate, and perfume. It is manufactured by Bode Chemie GmbH & Co., Hamburg, Germany.

### Treatment of hands and forearms

Human models with repetitive washing of the skin have been used both to estimate predictively the individual eczema risk [17] and to investigate the mildness and benefit of detergents and skin care products [18,19]. All subjects were divided into two groups (cross over design). One part of them (n = 13) washed hands and forearms with a standard neutral soap four times per day for two minutes each time over a period of 14 days. After a wash-out period of two weeks, they washed hands and forearms in the same way as before and applied in addition the cream after each hand wash to hands and forearms. The second part of the subjects (n = 12) did the same but in the opposite order.

### Test parameter

Irritant contact dermatitis due to detergents is reflected by scaling, erythema, and edema where the individual morphology depends upon dose, application time, and time of observation [20]. That is why the skin was assessed clinically. In addition, two objective parameter for skin irritation, skin roughness and hydration, were measured at the beginning of the study (baseline value) and at time day 2, 7, 9 and 14 of the study.

#### Skin roughness

The stratum corneum of the forearms was studied. Briefly, a silicon mass was disposed onto each of 12 sequential skin areas of the same size resulting in a cast of skin profile of an area of 12.5 cm^2^. The silicon masses were assessed with a 3D skin analyzer (Hommelwerke GmbH, Villingen-Schwenningen, Germany). The depth of the skin relief was measured for each sample and expressed as the mean roughness depth (RzDIN), according to the guidelines set in the German Industrial Standards (DIN). The analysis of the casts was restricted to six profile elments per sample, each 4 cm in length, and all sharing a common midpoint. The segments are defined by intervals in which the angle represents 30°. A damaged skin results in a higher RzDIN value [21,22]. Although the skin imprint technology has in some way limitations in figuring the finest structures of skin surface – because of shape of the tip of the mechanical stylus apparatus operating the imprints – nevertheless, this technology is highly relevant when comparing alterations in skin surface roughness before and after treatment with a cosmetic formula [23].

#### Skin hydration

The corneometer CM 820 (Courage & Khazaka Eletronic GmbH, Cologne, Germany) was used to measure skin hydration [24]. Measurements were done on the forearm and repeated 5 times for each skin area and time point. The arithmetric mean for each volunteer and time point was calculated. Measurements of skin hydration were accomplished under standardized room temperature uand humidity condtions (21.5°C, 45 RH), according to the recommendations of EEMCO [25].

### Data analysis

Statistical analysis for differences between treatment groups and time was conducted with the chi-square-test. A difference was considered as statistically relevant with a p < 0.05.

## Results

The clinical evaluation of the skin revealed that in both groups no visible eczema developed during the study period. Especially among subjects with a dry skin it was observed that despite extensive washing the skin condition appeared to be improved.

**Figure 1:**
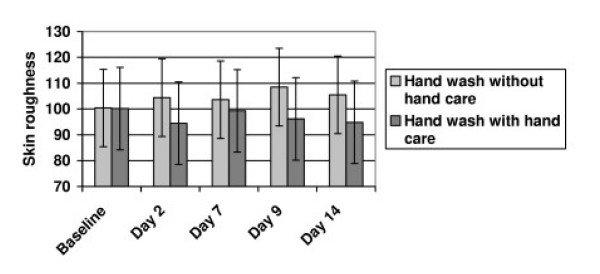
Mean skin roughness (and SD) measured on the forearms over two weeks; hand and forearms were washed daily 4 times for 2 min with a neutral soap, without hand care after the washes (group 1) and with a single application of a hand cream after each wash (group 2).

Baseline skin roughness before hand washing was 100.2 ± 19.7 in group 1 and 100.4 ± 16.9 in group 2. Washing hands for two minutes four times per day lead to a gradual increase of skin roughness to a maximum of 108.5 after 9 days (Figure 1). Use of a hand cream after each hand wash resultet in a decrease of skin roughness which the lowest means after 2 and 14 days (94.5 and 94.8, respectively). The difference of skin roughness between both groups was significant at each time point (p < 0.05; chi-square-test).

**Figure 2:**
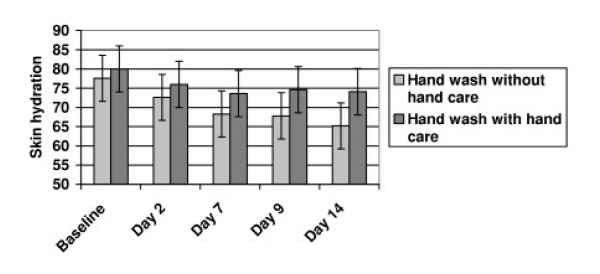
Mean skin hydration (and SD) measured on the forearms over two weeks; hand and forearms were washed daily 4 times for 2 min with a neutral soap, without hand care after the washes (group 1) and with a single application of a hand cream after each wash (group 2).

Baseline skin hydration before hand washing was 78.9 ± 8.7 in group 1 and 79.2 ± 10.2 in group 2. Washing hands for two minutes four times per day decreased skin hydration gradually to 65.5 after 14 days (Figure 2). The hand wash followed by use of a hand cream still decreased skin hydration after 2 days (76.1). Over the next 12 days, however, skin hydration did not change significantly (75.6 after 14 days). The difference of skin hydration between both groups was significant at each time point (p < 0.05; chi-square-test).

## Discussion

To our knowledge this is the first study of its kind to provide evidence that skin dryness and skin roughness caused by frequent hand washing can be attenuated by regular use of a hand cream. The effect is most likely explained by two different components of the cream: oil and wax components which prevent to some extent evaporation of epidermal water, and polyalcohols such as glycerol and propylen glycol which have moisturizing capacities on their own. Although the difference between the two treatment groups is rather small it is nevertheless an indication that the early parameter of irritant contact dermatitis can be attenuated by regular use of a hand cream. Clearly, the alterations in skin roughness are of minor magnitude, but early changes in skin texture which can be monitored by reliable methods are good indicators for a developing irritant skin reaction, finally becoming clinically relevant. Irritant contact dermatitis is one of the main occupational diseases among HCW and usually presents with rough and scaly skin, erythema and burning sensation [1]. It is mainly explained by hand washing and appears to be more severe when hard water is used [26]. According to the CDC guideline for hand hygiene hand washing by HCW should be restricted to situations when hands are visibly soiled [15]. Before the CDC guideline was published in 2002, a study over seven years revealed that after a promotion campaign for hand hygiene the overall compliance in hand hygiene was 66%, with a proportion of 30% attributed to hand washing and a proprtion of 36% attributed to hand disinfection [27]. This ratio explains that HCW do both in clinical practice, hand washing and hand disinfection. In order to reduce skin irritation by frequent hand washing it has been recommended in the USA that "HCW should be provided with hand lotions or creams to minimize the occurrence of irritant contact dermatitis associated with hand antisepsis or hand washing" [15]. An even more stringent recommendation has been issued in Germany in which hand care is described as a professional duty for HCW based on the perception that non-intact skin may serve as a reservoir for nosocomial pathogens and that hand disinfection may be less effective on skin which is not looked after [28].

In the current study each hand wash was followed by use of the cream in order to be able to measure the systemic effect of the two different treatment schemes. In clinical practice the pattern of use will certainly be different which is a limitation of our study. First of all, HCW will also apply quite frequently an alcohol-based hand rub to their hands. Commonly used alcohol-based hand rubs contain emollients and do not alter skin hydration [29]. In addition, use of a hand cream or lotion will probably not be possible after each hand wash "in real life" as it was done in our study. We have not studied occasional use of the cream which may resemble the clinical situation much better. But it appears to be valid to say that hand care is capable of attenuating skin dryness and skin roughness and that it is more important in clinical areas in which hand washing is done frequently.

In the CDC guideline for hand hygiene it is recommended that use of a skin care preparation should not impair the efficacy of hand antiseptics which may be applied after a cream or a lotion [15]. In addition it has been described as widely accepted that moisturizers are applied to affected skin for supporting the regeneration of the skin barrier as a secondary prevention of occupational hand dermatitis [30,31] although its efficacy is not confirmed [1]. But it appears to be clinically relevant to mention that the type of hand antiseptic and the type of skin care preparation should be selected wisely. For a hand wash preparation based on 4% chlorhexidine gluconate it has been shown before that its efficacy on the resident hand flora is completely abolished if a preparation containing anionic detergents (hand wash or hand care) is applied after the antiseptic agent [32]. It has also been shown before with three commonly used alcohol-based hand rubs that the efficacy on artificially contaminated hands is not impaired if two types of skin care preparations (oil in water, water in oil) are applied to the hands immediately before they are contaminated and treated with a hand disinfectant [33]. Although this type of evidence for alcohol-based hand rubs exists only for two skin care formulation it still indicates that the potential of skin care preparations to reduce the efficacy of alcohol-based hand rubs is low.

## Conclusion

Repetitive and frequent hand washing induces irritant contact dermatitis which increases with time and results in dry and rough skin. Use of a hand cream immediately after each hand wash can prevent both skin dryness and skin roughness. Regular use of skin care preparations should therefore help to reduce the main clinical symptoms of irritant contact dermatitis among healthcare workers in clinical practice.

## Competing interests

GK is a paid employee of Bode Chemie GmbH & Co., Hamburg, Germany. JE is a paid employee of Beiersdorf AG, Hamburg, Germany.

## Authors' contributions

GK analysed and interpreted the data and wrote the manuscript. JE coordinated the study and acquired the data. Both authors revised the article.

## Pre-publication history

The pre-publication history for this paper can be accessed here:
